# Supplementary material for: Copy Number Analysis of 9p24.1 in Classic Hodgkin Lymphoma Arising in Immune Deficiency/Dysregulation
Source: Cancers (Basel). 2024 Mar 27;16(7):1298. doi: 10.3390/cancers16071298 (PMC11011107; doi:10.3390/cancers16071298)
Supplement: Supplementary file 1 [file cancers-16-01298-s001.zip › cancers-2892495-supplementary.pdf]

A

PD-L1 H-Score

EBV	q1	q2	q3	q4
Negative	1	1	2	1
Positive	6	5	5	6

p=1

B

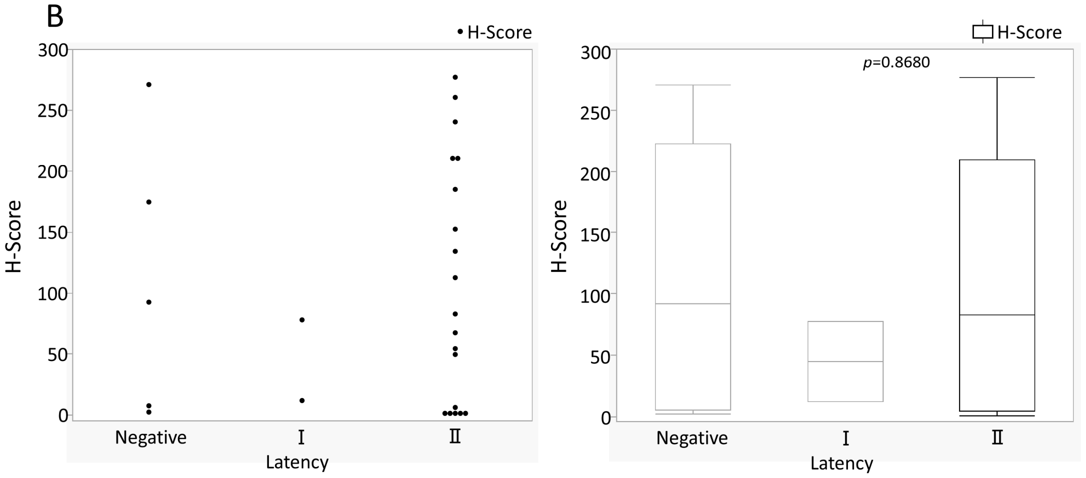

Figure S1: Relationship between EBV and H-Score in patients with CHL-IDD. (A) Distribution of EBV-negative and EBV-positive cases in PD-L1 immunohistochemistry (IHC) H-score quartiles; no correlation was found in the proportion of EBV-positive cases. (B) No correlation was found between EBV latency and H-Score.

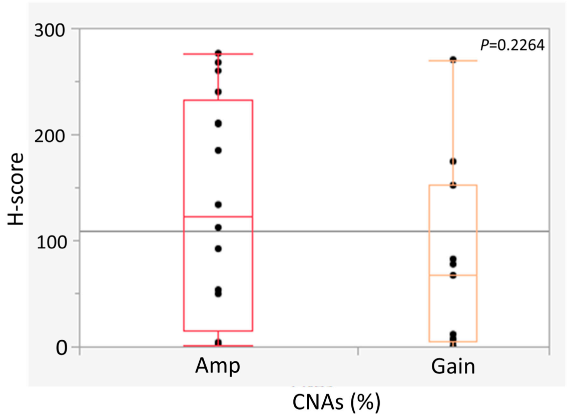

Figure S2: The association of PD-L1 IHC H-score and Amplification and Copy gain. No correlation was found between a copy gain group and an amplification group.

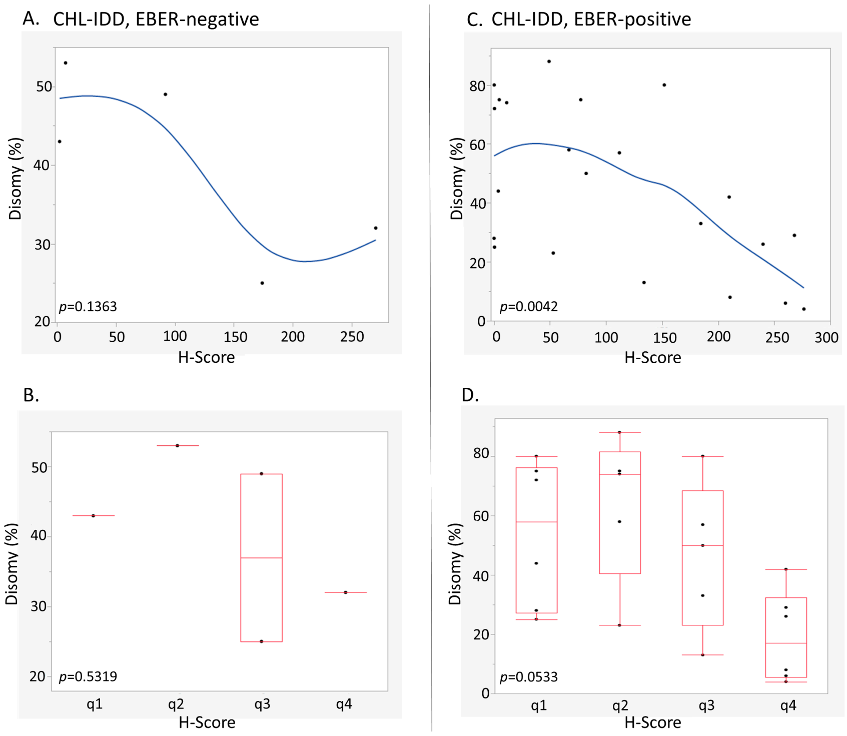

Figure S3: Relationship between H-score and 9p24.1 copy number alteration in patients with CHL-IDD. (A, B) Percentage of 9p24.1 residual disomic cells (y-axis) and PD-L1 IHC H-score (x-axis) plotted for individual EBV-negative and EBV-positive CHL-IDDs, respectively. (C, D) Percentages of residual 9p24.1 disomic cells in EBV-negative and EBV-positive cases in the respective PD-L1 IHC H-score quartiles from (A, B) were plotted. There was a trend toward an inverse correlation between H-Score and disomic cells in EBV-positive CHL-IDD cases but no inverse correlation in EBV-negative cases.

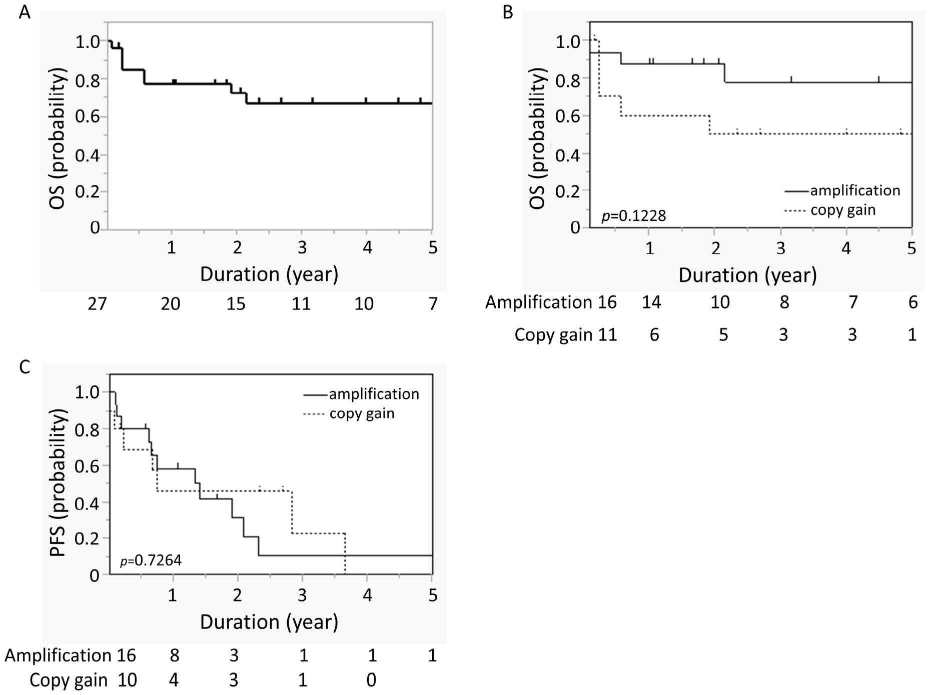

Figure S4: Kaplan-Meier curves in patients with CHL-IDD. (A) Overall survival (OS) for all patients. OS (B) and progression-free survival (PFS) for amplification and copy gain patients.

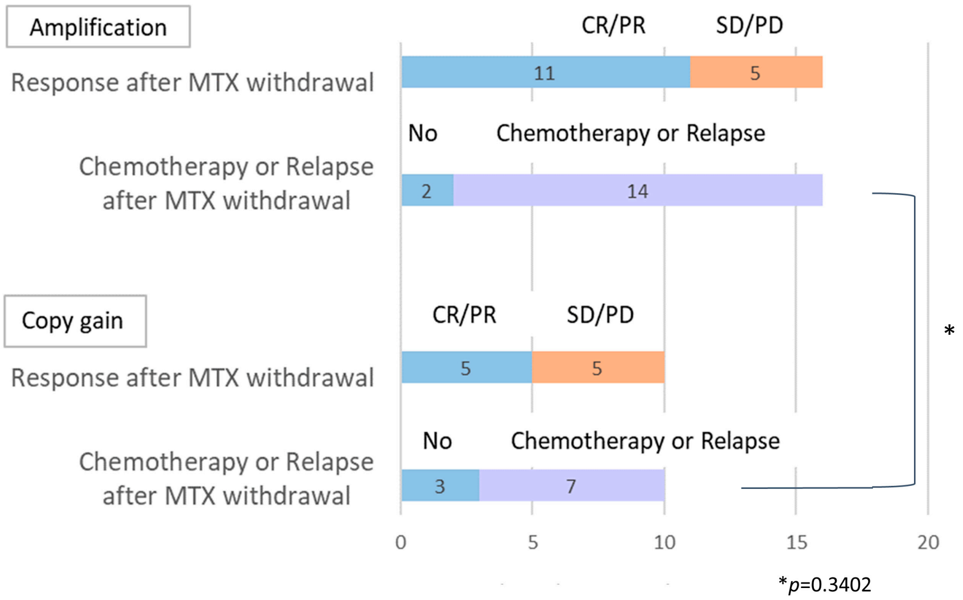

Figure S5: Changes in disease condition after MTX withdrawal in patients with CHL-IDD (copy gain and amplification). No significant differences in responses following MTX withdrawal and the need requirement for chemotherapy were observed between the amplification and copy gain groups.
